# Evaluation of surgical treatment of gout—A retrospective study on 28 cases with tophi

**DOI:** 10.1371/journal.pone.0313586

**Published:** 2025-01-24

**Authors:** Ting Zhang, Bin Yang, Xiaohong Xu, Zengfang Zhang, Zhenglun Pan

**Affiliations:** 1 Department of Rheumatology, Shandong University Qilu Hospital, China; 2 Qingdao Key Laboratory of Mitochondrial Medicine, China; 3 Department of Hand and Foot Surgery, Shandong University Qilu Hospital, China; University of Roehampton - Whitelands College, UNITED KINGDOM OF GREAT BRITAIN AND NORTHERN IRELAND

## Abstract

**Introduction:**

The efficacy, safety, optimal timing, and urate-lowering effects of surgical interventions in gout management remain poorly understood. This study aims to fill this gap by evaluating the role of surgery in treating gout patients with tophi.

**Method:**

A retrospective analysis was conducted on 28 gout patients presenting with tophi. Data were comprehensively retrieved from electronic medical records, including medical history, laboratory findings, surgical procedures, hospitalization duration, postoperative monitoring, and relevant variables.

**Results:**

Postoperative improvements were observed in joint symptoms and functionality. Surgical intervention effectively reduced the frequency of gout flares, demonstrating short-term urate-lowering effects (STULE) and potential long-term urate-lowering effects (LTULE) when combined with urate-lowering treatments (ULT). Primary healing occurred in 65 out of 67 surgical sites (97.01%), with only 2 sites (2.99%) experiencing delayed healing, and minimal complications reported. Prolonged hospital stays were associated with elevated leukocyte counts, C-reactive protein (CRP), and erythrocyte sedimentation rate (ESR) levels, as well as a higher number of surgical sites, rather than serum uric acid (SUA) levels.

**Conclusions:**

Surgical intervention is a promising and safe therapeutic option for managing gout, particularly in cases with joint deterioration, functional impairment, or nerve involvement. Surgery not only reduces the frequency of gout flares but also provides STULE and potential LTULE when complemented with ULT. Patients with lower inflammatory indices and fewer incisions exhibit faster postoperative recoveries. Optimal timing of surgery, ideally during periods of disease remission, is crucial for minimizing complications and reducing hospitalization durations.

## Introduction

Gout, a disease characterized by excessive urate levels due to purine metabolism disorder, often initially presents as acute gouty arthritis [1]. Without timely and effective treatment, chronic urate deposition in joints can lead to the formation of giant tophi [2]. Foot involvement is particularly common, with the first metatarsophalangeal joint and Achilles tendon being primary sites of monosodium urate deposition and tophi formation [3–6]. Hand involvement is also frequent [7–9]. Patients with tophi in the feet and hands frequently experience significant disability, impacting daily activities such as carrying objects and walking, and leading to functional impairments [10, 11].

While urate-lowering treatment (ULT) remains the cornerstone of gout management, certain cases necessitate additional interventions [11, 12]. Surgical removal of tophi may become essential in the presence of severe complications, including functional disability, infection, ulceration, joint destruction, or nerve involvement. However, comprehensive data on the efficacy, safety, timing, and urate-lowering potential of surgical intervention remain limited [12].

In this retrospective study, we analyzed 28 patients who underwent surgical removal of tophi between January 1, 2014, and December 31, 2021. Our objective was to reassess the efficacy, safety, timing, and potential urate-lowering effects of surgical intervention within the broader context of gout management. This analysis provides valuable insights into the role of surgery in treating advanced gout and highlights critical considerations for optimizing patient outcomes.

## Patients, materials, and methods

### Patients and materials

All patients included in this study met the 2015 ACR/EULAR gout classification criteria. Detailed clinical data were collected based on stringent inclusion and exclusion criteria.

#### Inclusion criteria

1. Serum urate (SUA) levels of > 420 µmol/L (7.0 mg/dL) in males and > 350 µmol/L (6.0 mg/dL) in females, either previously or currently. 2. A history of joint gout characterized by episodes of redness, swelling, heat, and pain, with symptoms resolving post-attack. 3. Presence of tophi affecting joint function or a patient request for the removal of large tophi. 4. No surgical contraindications. 5. Postoperative pathological examination confirming the presence of urate crystals.

#### Exclusion criteria

1. Postoperative pathological findings inconsistent with the diagnosis of gout. 2. Incomplete follow-up. 3. Presence of other severe diseases or treatments significantly affecting SUA levels.

#### Surgical method

Anesthesia methods varied according to patient conditions, with options including brachial plexus block anesthesia, continuous epidural anesthesia, or general anesthesia. The surgical procedure involved several critical steps: excising ulcerated skin, enlarging the incision, thoroughly removing urate crystals, and repeatedly washing the wound with 5% sodium bicarbonate saline using a pulse gun. Efforts were made to preserve unaffected tendon and peritendon tissue to prevent postoperative tendon adhesion. Additionally, eroded joint capsules, tendons, cartilage, and cancellous bone were removed. Arthrodesis was performed for severely damaged interphalangeal joints, and tendons and ligaments were repaired. Finally, resected tissues were sent for pathological examination.

The detailed manifestations of patients were meticulously recorded and accessed for research purposes on May 1,2023, including previous medical history, laboratory examination results, surgical method and location, length of hospital stay, prognosis, postoperative follow-up, and other relevant information. Authors did not have access to information that could identify individual participants during or after data collection. No data that could identify single patients are presented therefore signed informed consent was not needed prior to inclusion.

### Statistical methods

Categorical data were presented as percentages, while continuous data were expressed as the mean ± standard deviation (SD) or median (range). The normality of the variables was assessed using the Shapiro–Wilk test.

To compare the efficacy of urate-lowering therapy (ULT) post-operation, including serum urate (SUA) levels, incidence of recurrent arthritis (episodes per year), and new-onset arthritis (episodes per year) between the standard ULT group and the non-standard ULT group, Student’s t-test was utilized. The average length of hospital stay was compared across different groups categorized by preoperative SUA level, white blood cell count (WBC), erythrocyte sedimentation rate (ESR), C-reactive protein (CRP), and the number of operation sites using Student’s t-test.

Analytical results were reported as crude odds ratios (OR) with 95% confidence intervals (CI). All statistical tests were two-sided, with a significance threshold set at P < 0.05. Statistical analyses were conducted using SPSS software version 19.0 (SPSS Inc., Chicago, IL, USA).

### Ethics approval and consent to participate

This study was approved by the Institutional Medical Ethics Review Boards of Qilu Hospital of Shandong University (Qingdao). This study was performed in accordance with the ethical standards laid down in the 1964 Declaration of Helsinki and its later amendments.

No data that could identify single patients are presented therefore signed informed consent was not needed prior to inclusion.

## Results

### General information (Table 1)

**Table 1:** General information of the 28 cases underwent surgery.

NO	Sex	Age	hospital stay(day)	UA(mmol/L)	UA(one day after surgery,mmol/L)	CRP(mg/L)	ESR(mm/h)	WBC(*10^9)
1	M	39	7	599	433	1.23	2	6.22
2	M	41	10	614	476	4.29	13	8.09
3	M	40	9	468	369	6.23	7	9.49
4	M	65	11	354	331	6.23	34	5.1
5	M	73	12	756	544	6.7	4	9.31
6	M	38	15	431	578	7.47	22	6.39
7	M	35	19	445	439	7.49	19	7.16
8	M	41	9	519	467	8.33	16	7.08
9	M	58	20	526	467	9.07	24	4.69
10	M	41	11	524	477	10.22	23	4.43
11	M	51	7	446	344	11.02	5	5.55
12	M	79	23	571	489	11.28	29	4.54
13	M	68	25	655	439	12.01	18	4.03
14	M	63	5	611	552	12.27	4	8
15	M	54	11	654	435	12.32	26	10.11
16	M	41	12	526	349	12.49	43	7.69
17	M	30	18	651	475	15.03	32	7.1
18	M	61	17	579	423	17.3	32	3.99
19	M	53	22	492	454	17.55	17	5.02
20	M	44	9	387	422	22	3	3.1
21	M	48	7	573	432	22.39	2	13.77
22	M	61	11	655	626	22.97	22	15.62
23	M	37	28	382	345	23.59	17	6.12
24	M	61	14	560	440	25.57	11	9.52
25	M	27	14	820	536	32.67	36	5.6
26	M	38	10	345	401	52.13	24	9.27
27	M	66	10	780	466	81	43	3.65
28	M	49	18	616	435	138.21	18	4.66

All 28 patients included in this study met the 2015 ACR/EULAR gout classification criteria. The average age of the patients was 50.07 years, with a range of 27 to 79 years. Clinical presentations included swelling and stiffness of the involved joints. Among the patients, 22 (78.6%) experienced functional impairment or joint instability, 2 (7.1%) suffered from nerve compression, and 4 (14.3%) had difficult-to-heal skin ulcers. Additionally, three patients had renal calculi, although none presented with renal function failure. The mean preoperative serum urate (SUA) level was 554.96 µmol/L, with a range of 345 to 820 µmol/L.

#### Preoperative management

All patients underwent comprehensive preoperative management, which included dietary adjustments to reduce high purine intake. Additionally, patients were advised to avoid fatigue and cold, maintain a calm state of mind, and ensure daily urine output exceeded 2500 ml. These measures were implemented to optimize patient condition and minimize potential complications during surgery.

#### Postoperative management

Serum urate (SUA) levels were monitored monthly. During acute gout flares, patients received colchicine or non-steroidal anti-inflammatory and analgesic drugs. Additionally, patients underwent rehabilitation and physiotherapy to aid recovery and improve joint function. Urate-lowering therapy (ULT) was initiated postoperatively to maintain reduced urate levels and prevent further crystal formation.

### Surgical sites and tophi characteristics

**Fig 1:**
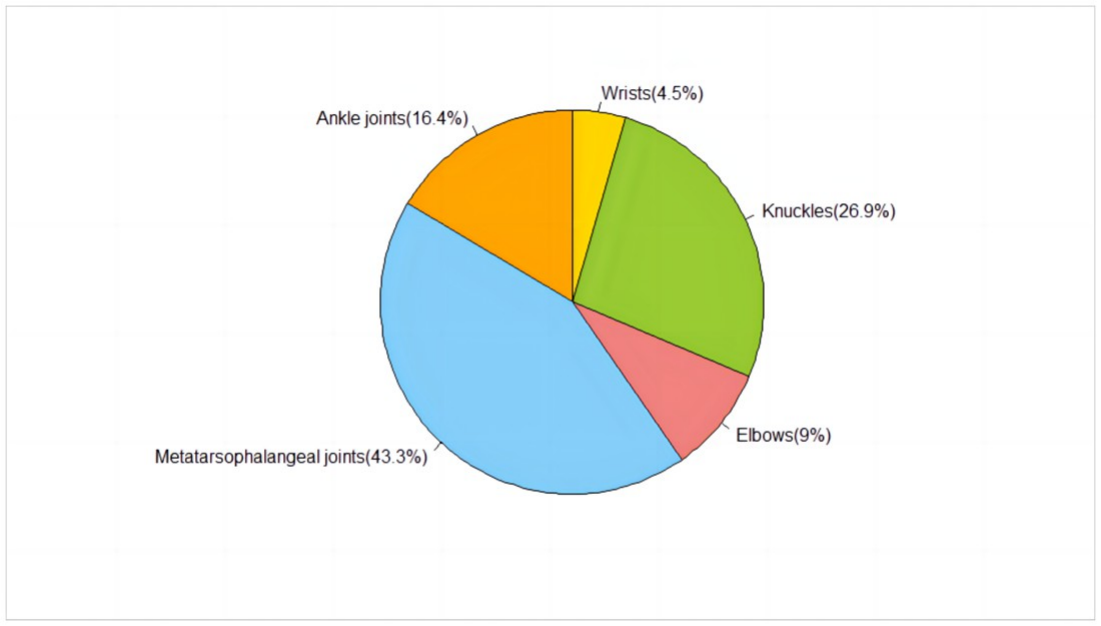
Distribution of surgical sites among patients.

A total of 67 surgical sites were addressed among the 28 patients (Fig 1). The size of the tophi varied, ranging from 1.5 cm × 1.0 cm × 1.0 cm to 5.4 cm × 4.6 cm × 4.8 cm.

### Imaging assessments and findings

**Fig 2:**
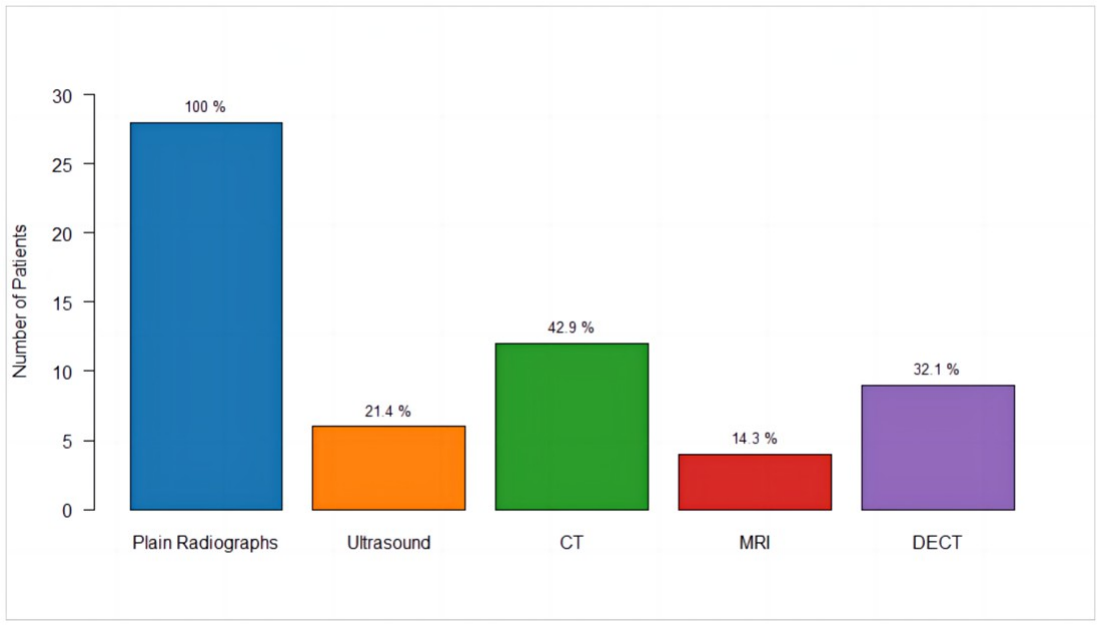
Distribution of imaging modalities among patients.

Plain radiographs, computed tomography (CT), ultrasound, magnetic resonance imaging (MRI) and Dual-energy computed tomography (DECT) were frequently performed on patients with tophus gout (Fig 2). These imaging techniques provided comprehensive assessments of the extent and nature of tophi and associated joint damage.

**Fig 3:**
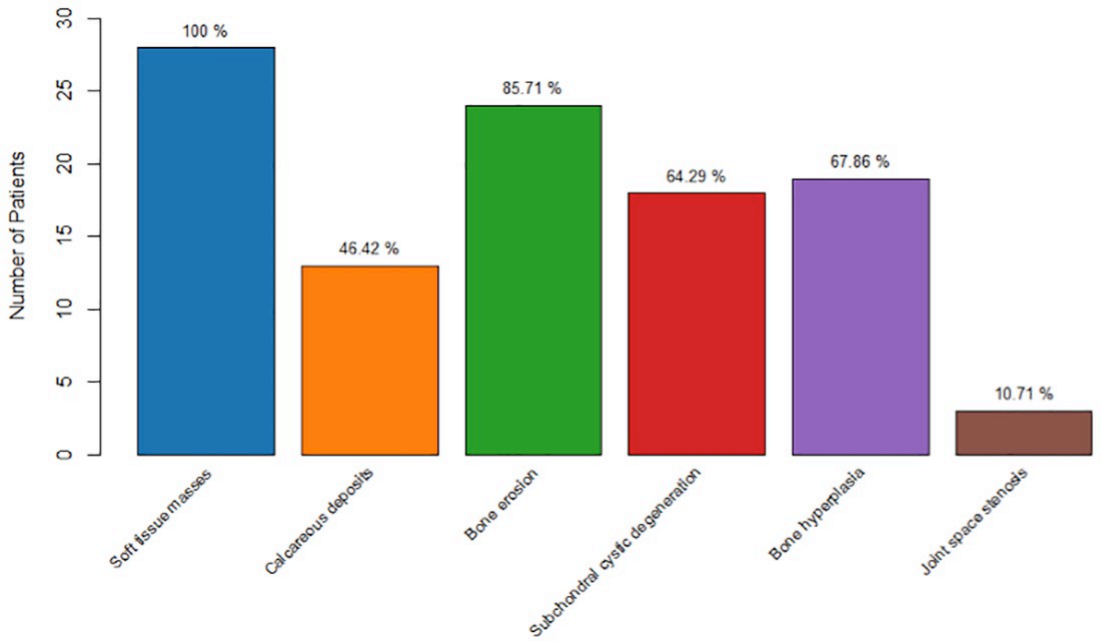
The primary imaging manifestations.

The primary imaging manifestations of gout included soft tissue masses presenting as eccentric nodular protrusions (28 patients), calcareous deposits (13 patients), bone erosion (24 patients), subchondral cystic degeneration (18 patients), bone hyperplasia (19 patients), and joint space stenosis (3 patients) (Fig 3). These findings provided critical insights into the extent of tophaceous deposits and the degree of joint and bone involvement.

## Surgical related results

### Postoperative wound healing

All 28 cases were followed up for a period ranging from 6 to 48 months. Among the 67 surgical sites, 65 sites (97.01%) achieved primary healing, while 2 sites (2.99%) experienced delayed healing. This high rate of primary healing underscores the effectiveness of the surgical intervention and postoperative management in this patient cohort.

### Efficacy of operation

Patients were followed up for a minimum of 6 months. Joint pain completely disappeared in 26 cases and was significantly relieved in 2 cases. All patients experienced satisfactory improvement in joint function, and no serious complications were reported. This indicates a high success rate in alleviating symptoms and enhancing the quality of life for patients following surgical intervention.

### Serum urate levels and long-term outcomes

The serum urate (SUA) levels measured three days before the operation averaged 554.96 ± 99.34 µmol/L. One day post-operation, the SUA levels significantly decreased to 451.57 ± 79.43 µmol/L, a difference that was statistically significant (P = 0.034).

As of December 1, 2023, significant differences in SUA levels, the incidence of recurrent arthritis, and the incidence of new onset arthritis were observed between the 22 patients who maintained ULT (diet and drug) and the 6 patients who did not follow ULT post-operation. Compared to preoperative conditions, both the incidence of flares and SUA levels markedly decreased in the ULT group, whereas in the non-ULT group, only SUA levels showed a reduction. Detailed comparisons are presented in Table 2.

**Table 2:** Efficacy of ULT in 6 months after operation.

Group	SUA (umol/L)		Incidence of recurrent arthritis (times/year)		New onset arthritis (times/year)	
	BO	AO	BO	AO	BO	AO
**Standard ULT (22)**	546.32± 82.76	416.57±79.43*^P1✮P1^	4.1±0.8	0.9±0.4*^P2✮P2^	1.9±0.3	0.6±0.2*^P3✮P3^
**Non-Standard ULT (6)**	567.28 ±92.36	523.79±103.62^✮P4^	3.8±0.6	3.6±1.0	2.2±0.2	2.1±0.9

*P<0.05 (Standard ULT group vs Non-Standard ULT group, *P1 = 0.037, *P2 = 0.018, *P3 = 0.044)

^✮^**P<0.05(**Before operation vs After operation, ^✮^P1 = 0.022, ^✮^P2 = 0.041, ^✮^P3 = 0.031, ^✮^P4 = 0.047)

BO: Before operation; AO: After operation;

### Factors affecting hospitalization stay

The hospitalization time was significantly shorter for patients with a leukocyte count (3.5–9.5×10^9/L) ≤ 9.5 × 10^9/L, C-reactive protein (CRP) (0–8 mg/L) ≤ 8 mg/L, and erythrocyte sedimentation rate (ESR) (0-15mm/h) ≤ 15 mm/h compared to those with higher levels. Specifically, patients with a leukocyte count ≤ 9.5 × 10^9/L had an average hospital stay of 10.62 days versus 14.25 days for those with counts > 9.5 × 10^9/L (P = 0.045). Patients with CRP levels ≤ 8 mg/L stayed an average of 11.36 days compared to 14.23 days for those with CRP > 8 mg/L (P = 0.039), and those with ESR ≤ 15 mm/h stayed 8.89 days compared to 14.75 days for those with ESR > 15 mm/h (P = 0.020). Furthermore, patients with only one surgical site had significantly shorter hospital stays (10.13 days) compared to those with multiple surgical sites (13.20 days, P = 0.041). However, SUA levels at admission did not significantly affect the length of hospital stay (P = 0.072) (Table 3).

**Table 3:** Factors that influence the length of hospital stay.

Factors	Group	Average length of hospital stay (day)	P value
**UA**	≤420umol/L	12.50	0.072
	>420umol/L	12.67	
**WBC**	≤9.5*10^9/L	10.62	0.045
	>9.5*10^9/L	14.25	
**CRP**	≤8mg/L	11.36	0.039
	>8mg/L	14.23	
**ESR**	≤15mm/h	8.89	0.020
	>15mm/h	14.75	
**Number of operation site**	1	10.13	0.041
	≥2	13.20	

## Discussion

In this study, we evaluated the safety, effectiveness, timing, and potential urate-lowering role of surgical intervention within the comprehensive treatment landscape for gout. Urate-lowering therapy (ULT) remains the cornerstone of gout management; however, approximately 12–35% of gout patients develop tophi despite ULT [13]. Achieving ULT goals in these patients can be challenging due to insufficient dosage, short treatment duration, or intolerable adverse drug reactions. In cases of severe complications such as infection, ulceration, joint destruction, loss of function, or nerve involvement, ULT alone may be insufficient, necessitating surgical intervention as a last resort [14].

The primary aim of surgery in most of our cases was to restore or improve function, reflecting the significant impact of foot and hand involvement on patient functionality compared to other joints such as the elbow or knee. The attitude towards surgical intervention has evolved positively since 1943 when Linton RR and Talbott JH [15] proposed initial indications for surgery. By 2016, Kasper IR [12] outlined more comprehensive surgical indications, including refractory disease despite maximal medical therapy, poorly controlled pain attributed to tophi, nerve compression or entrapment, uncontrolled or recurrent infection, discharging sinus, significant skin ulceration, functional impairment, joint instability, and impaired joint motion preventing work or daily activities.

The expansion of surgical indications reflects advancements in medical care. In our study, surgical treatment for tophi proved relatively safe, with 65 of 67 surgical sites (97.01%) achieving primary healing and only 2 sites (2.99%) experiencing delayed healing. This contrasts with published data indicating a major complication rate of 53% for delayed healing [16]. Factors such as peripheral vascular disease, diabetes, or steroid use likely increase postoperative complications. Our cohort experienced no serious complications.

Postoperatively, serum urate levels decreased significantly on the first day, indicating a short-term urate-lowering effect (STULE) associated with surgery [16–18]. STULE likely results from both the removal of tophi, reducing urate burden, and surgery-induced inflammation, which enhances renal urate excretion during acute inflammatory states [19]. Additional factors such as fasting, intravenous fluid administration, and other variables may also contribute to STULE. Interestingly, our data showed that even six patients who did not maintain ULT postoperatively had lower SUA levels six months after surgery, partially contradicting the view that surgery lacks significant long-term impact on SUA [20, 21]. Surgery can reduce urate burden and prompt patients to adhere to a low-purine diet and appropriate functional exercise. The 22 patients who maintained standard ULT had significantly lower SUA levels than the six who did not, underscoring the benefits of continued ULT post-surgery. The long-term urate-lowering effect (LTULE) associated with surgery can be achieved through sustained ULT.

Our data also suggested that gout surgery reduced the frequency of acute gout flares. While ULT likely contributes to this reduction, the "urate burden reduction" effect from surgery should not be overlooked. The 22 patients who maintained ULT experienced fewer gout flares and new-onset tophi compared to the six patients without ULT, highlighting ULT’s importance in gout treatment.

Leukocyte count, ESR, and CRP levels correlated with the degree of joint inflammation and infection, impacting incision healing. Increased leukocyte, CRP, and ESR levels, as well as the number of surgical sites, were associated with prolonged hospital stays, rather than elevated SUA levels. This suggests that performing surgery during the remission stage of gout can help reduce complications and hospital stay durations, and that anti-infection treatment is necessary for patients with skin infections [22].

Despite the valuable insights provided by our study, several limitations must be acknowledged. The relatively small sample size of 28 patients limits the generalizability of the results, necessitating larger, multi-center studies to validate these findings across diverse populations. The retrospective design restricts our ability to establish causality, indicating the need for prospective, randomized controlled trials. The follow-up duration for some patients was relatively short, so longer-term outcomes and the potential recurrence of tophi require further investigation. Adherence to urate-lowering therapy (ULT) postoperatively varied, potentially confounding results and underscoring the importance of consistent ULT adherence. While we identified peripheral vascular disease, diabetes, and steroid use as contributors to postoperative complications, other unmeasured confounders may have influenced outcomes. The mechanisms underlying short-term urate-lowering effects (STULE) and long-term urate-lowering effects (LTULE) post-surgery were not fully elucidated, requiring further exploration. Lastly, the optimal timing for surgical intervention in gout patients remains unclear, and additional research is needed to determine the best timing to minimize complications and maximize therapeutic outcomes. These limitations highlight the need for further research to confirm our findings and optimize treatment strategies for patients with refractory gout.

## Conclusions

Surgery can be a safe and effective option for treating gout complications such as joint destruction, functional impairment, and nerve involvement. Surgical intervention may reduce the frequency of gout flares and is associated with short-term urate-lowering effects (STULE). When combined with urate-lowering therapy (ULT), there is potential for long-term urate-lowering effects (LTULE). Patients with lower inflammation indices and fewer surgical sites tend to recover more rapidly from surgery. Optimal timing for surgery is during periods without gouty flares to minimize complications and shorten hospital stays. While our study provides important insights into the safety and efficacy of surgical interventions in gout management, these limitations highlight the need for further research to confirm our findings and optimize treatment strategies for patients with refractory gout.

## Supporting information

S1 Data (XLSX)

S1 Checklist STROBE statement—Checklist of items that should be included in reports of observational studies. (DOCX)
